# A notes bivariate power law processes: conditional intensity and parameter estimation techniques^,^^☆^^☆☆^

**DOI:** 10.1016/j.mex.2026.104018

**Published:** 2026-06-29

**Authors:** Andi Kresna Jaya, Nurtiti Sunusi, Erna Tri Herdiani

**Affiliations:** Stochastics Modelling Research Group, Department of Statistics, Faculty of Mathematics and Natural Sciences, Hasanuddin University, Jl. Perintis Kemerdekaan km 10, Kampus Tamalanrea, Makassar, South Sulawesi, 90245, Indonesia

**Keywords:** Point process, Poisson process, Non-homogeneous, Bivariate, Conditional, Intensity function

## Abstract

The point process model effectively represents the number of random events occurring over time through its intensity function. When events are of two types, a bivariate point process allows simultaneous analysis of each event’s intensity. This study develops a conditional intensity model for a non-homogeneous bivariate point process over time with an event rate approach that follows a certain pattern over time in the form of a time-dependent power law intensity function with two parameters, an initial intensity parameter and a control parameter governing the change in the event rate over time. Parameter estimation is performed using the maximum likelihood method derived from the probability of one event occurring in a very short interval and the non-occurrence at other times. The results of the analysis show that:

• The effect of observation duration on model parameters is not linear but depends on its interaction with the pattern of changes in the event rate over time.
• The higher the number of events observed, the higher the estimate of the initial intensity of the event.
• Both the duration of observation and the timing of events contribute significantly to determining the rate at which the event rate changes over time.

## Graphical abstract

**Figure:**
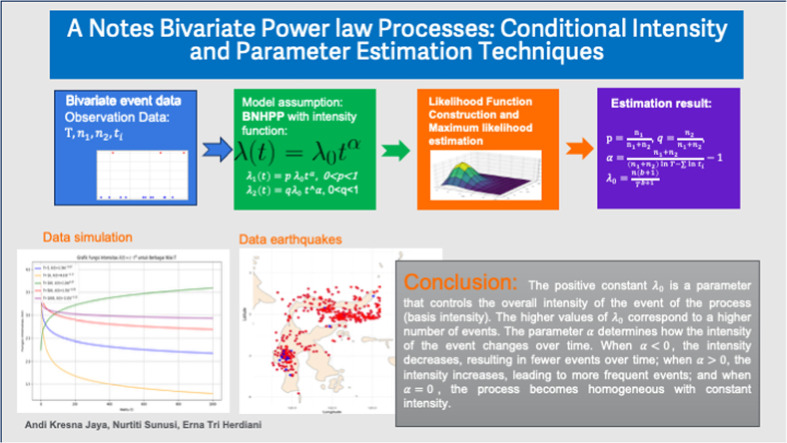

**Specifications table**

**Table:** 

**Subject area**	Mathematics and Statistics
**More specific subject area**	**Probability and Stochastics Processes**, we study processes that evolve over time and developed a conditional intensity model with time-dependent parameters for bivariate non-homogeneous Poisson processes.
**Name of your method**	The Bivariate non-homogeneous Poisson process maximum likelihood method
**Name and reference of original method**	None
**Resource availability**	None

## Background

The point process is a powerful modelling framework for describing sequences of events that occur irregularly in time or space. Random events can be represented as point distributed stochastically. In epidemiology, for instance, point processes are used to study the spread of diseases, especially those classified as extraordinary events occurring within specific regions and time frames. In seismology, as discussed in [[1], [2], [3]], earthquake occurrences are modelled as random events using point processes. The Poisson process itself is a special case within the broad class of point processes, as shown in [[4], [5], [6]].

The Poisson process models the number of random events in time or space, assuming independent and exponentially distributed time intervals between events. If the event rate is constant, it is called a homogeneous Poisson process (HPP), when time-dependent, it becomes a non-homogeneous Poisson process (NHPP).

The NHPP is widely used due to its flexibility in modelling varying event rates. Examples include earthquake seismic data [2], epidemic spread [7] and spatial point processes [8]. These studies demonstrate how NHPP extends the Poisson process by allowing the event intensity to vary over time or space, making it suitable for real-world phenomena with dynamic patterns.

These studies consider the events to be single events and model them as NHPP models with one variable. Although this model provides information on the randomness of events, the complexity of the phenomenon may not include interactions between events as separable variables [9]. For example, in seismological studies, aftershocks are often associated with the main shock [2] and [10]. Meanwhile, in epidemiology, the spread of disease in one location may be influenced by the distribution pattern in surrounding locations [7]. The various univariate NHPP models discussed in [11] are inadequate to describe the relationship between groups of events because they remain limited to a single event variable. Therefore, [12] or [13] have raised the issue of two or more Poisson processes events for further study. The bivariate point process approach to grouping two different events of a point process becomes relevant to capture this relationship. By grouping events into two types, the process allows for richer analysis of complex patterns emerging in the data and can even suppress the effect of non-convergence in the parameter estimators in the formation of its likelihood function.

This research aims to extend the concept of the bivariate point process developed by [14] for the HPP to the NHPP form. This extension of the concept is expected to provide a more comprehensive solution for the analysis of random phenomena involving two groups of events with variable intensity. In addition to deriving the conditional intensity function and maximum likelihood estimators, the adequacy of the proposed model is assessed through a goodness of fit based on the time rescaling theorem by [15] This diagnostic approach evaluates whether the transformed inter-event times are consistent with and Exponential distribution with parameter λ=1.

## Method details

Suppose that time is expressed as a space in which random events that have occurred and those that may occur are placed within that time dimension. The distribution appears random and the next event point is also uncertain. Realizing the distribution pattern of the locations of the points that have occurred will provide information about the next possible event. If the observed event points are evenly distributed randomly over the observation time interval, they can be modeled as an HPP [16]. If event points appear unevenly distributed, an NHPP model can be a solution.

## Non-homogeneous Poisson process

The Poisson process model has an intensity of the event at a constant rate λ with the time between events following an exponential distribution. For event intensity to be a time-dependent function λ(t), the time between events no longer follows an exponential distribution. Observing the number of events in time t≥0 is a stochastic process and is expressed as a counting process.

Definition 1 Let N(t) denote the number of events in time t. {N(t),t≥0} is called a counting process if N(0)=0 and N(t+s)≥N(s) for all s≥0.

Suppose that the number of events in interval (t,t+s] is N(t+s)−N(s) and the number of events in the interval (0,t] is N(t). The number of events in these two intervals is independent. A counting process that satisfies this property is called an independent increment.

Definition 2 The counting process {N(t),t≥0} is called a non-homogeneous Poisson process with intensity rate λ(t), which is a process with independent increments for different time intervals and has a Poisson distribution with mean parameter function $\Lambda \left(t\right)=\int_{0}^{t}\lambda \left(s\right)ds$.

The number of random events in the time interval (0,t] has Poisson distribution, means its probability function,

(1) $$P\left(N\left(t\right)=n\right)=\frac{{\left(\Lambda \left(t\right)\right)}^{n}}{n!}\exp\left(-\Lambda \left(t\right)\right).$$

This form of the probability function in Eq. (1) states that there is a probability that exactly n events will occur in the time interval (0,t]. The probability that no events will occur in the interval (0,t], N(t)=0,

(2) $$P\left(N\left(t\right)=0\right)=\exp\left(-\int_{0}^{t}\lambda \left(s\right)ds\right).$$

The expression Eq. (2) indicates that the time between events follows a probability function of the form

(3) $$f\left(t\right)=\lambda \left(t\right)\exp\left(-\int_{0}^{t}\lambda \left(s\right)ds\right).$$

Suppose that observation for events in the process for (0,T] yield N observed events. The first, second, and so on up to the N^th^ event are observed. Suppose that in the time subinterval $\left(0,t_{1}\right]$, first event occurs, and second event occurs within the interval $\left(t_{1},t_{2}\right]$ and so on until N^th^ event occurs in the interval $\left(t_{N-1},t_{N}\right]$. For each i=1,2,⋯,N, an event occurs in a very short time of Δ (Δ→0), and no others events occur in any other time interval within the observation time (0,T]. The probability of a single event occurring within the interval Δ, without other events occurring in the remaining portion of the interval $\left(t_{i-1},t_{i}\right]$ is obtained by multiplying the probability given in Eq. (2) and Eq. (3). This yields the following expression:

$$P\left(N\left(t_{i-1},t_{i}\right]=1\right)=\lambda \left(t_{i}\right)\Delta \exp\left(-\int_{t_{i-1}}^{t_{i}}\lambda \left(t\right)dt\right).$$

The probability that no events occur during the remaining observation interval $\left(t_{N},T\right]$ is given by

$$P\left(N\left(t_{N},T\right]=0\right)=\exp\left(-\int_{t_{N}}^{T}\lambda \left(t\right)dt\right).$$

Therefore, for the entire observation time (0,T] and there are N observation points, the likelihood function is obtained

$$L\left(\lambda \left(t\right);t_{1},t_{2},\cdots ,t_{N},\;T\right)=\left(\prod_{i=1}^{N}P\left(N\left(t_{i-1},t_{i}\right]=1\right)\right)\times P\left(N\left(t_{N},T\right]=0\right)$$

$$=\left(\prod_{i=1}^{N}\lambda \left(t_{i}\right)\Delta \exp\left(-\int_{t_{i-1}}^{t_{i}}\lambda \left(t\right)dt\right)\right)\times \exp\left(-\int_{t_{N}}^{T}\lambda \left(t\right)dt\right)$$

$$=\left(\prod_{i=1}^{N}\lambda \left(t_{i}\right)\right)\Delta^{n}\exp\left(-\int_{0}^{T}\lambda \left(t\right)dt\right).$$

## Event intensity

The function Λ(t) in Definition 2 is called the mean function of the non-homogeneous Poisson distribution, while λ(t) is called the intensity function for NHPP. The intensity function or event intensity is the average rate at which events are expected to occur in a unit of time given the history of previous events. This intensity function serves to regulate the rate of events that change over time. The counting process {N(t),t≥0} has an intensity function that depends not only on time t but also on the history of the process itself. Suppose $H_{t}$ denotes the history of the process up to time t, then its intensity function is expressed in the general form $\lambda (t|H_{t})$.

According to Eq. (3), the conditional intensity function serves as a parameter function for the probability distribution of inter-event times (inter-arrival times). Based on this relationship, a NHPP can be constructed from the distribution of inter-event times. If the conditional intensity function is constant, $\lambda (t|H_{t})=\lambda$, then the process reduces to a HPP, where events occur at λ, a constant rate over time. In this case, the mean function is given by Λ(t)=λt, and the probability distribution of the number of events N(t) follows a Poisson distribution:

$$P\left(N\left(t\right)=n\right)=\frac{{\left(\lambda t\right)}^{n}}{n!}\exp\left(-\lambda t\right).$$

The intensity function $\lambda (t|H_{t})$ is nonnegative and must be well integrated for t→∞. The intensity function can be expressed as the ratio of the probability function of the inter-event times to the survival function. As an example, a HPP has an inter-event time distribution given by the probability density function f(t)=λexp(−λt), which correspond to the exponential distribution with the rate parameter λ. The corresponding survival function is $S\left(t\right)=1-\int_{0}^{t}f\left(s\right)ds$. Then the conditional intensity function is given by

$$\lambda \left(t|H_{t}\right)=\frac{f\left(t\right)}{S\left(t\right)}=\frac{\lambda \exp\left(-\lambda t\right)}{1-\int_{0}^{t}\lambda \exp\left(-\lambda s\right)ds}=\frac{\lambda \exp\left(-\lambda t\right)}{1-\left(1-\exp\left(-\lambda t\right)\right)}=\frac{\lambda \exp\left(-\lambda t\right)}{\exp\left(-\lambda t\right)}=\lambda .$$

For the inter-event time, there are two special distributions that are closely related to the exponential distribution. The first is the Gamma distribution, parameterized by shape α and rate β with the probability density function

$$f\left(t\right)=\frac{t^{\alpha -1}}{\Gamma \left(\alpha \right)\beta^{\alpha }}\exp\left(-\frac{t}{\beta }\right).$$

The second is the two-parameter Weibull distribution with probability density function

$$g\left(t\right)=ct^{b}\exp\left(-\frac{ct^{b+1}}{b+1}\right).$$

When the inter-event time follows a Gamma distribution with shape parameter α=1 and scale parameter β=1/λ, it reduces to the exponential distribution with rate λ. Similarly, when the inter-event time follows a Weibull distribution with parameter b=0 dan c=λ, the distribution also simplifies to the exponential distribution with rate λ.

Let t denote the inter-event time, which follows a Weibull distribution. In this model, the scale parameter c>0 and the shape parameter b such that −1<b, corresponding to a NHPP. The conditional intensity function is given by:

(4) $$\lambda \left(t|H_{t}\right)=\frac{g\left(t\right)}{S\left(t\right)}=\frac{ct^{b}\exp\left(-\frac{ct^{b+1}}{b+1}\right)}{1-\int_{0}^{t}cs^{b}\exp\left(-\frac{cs^{b+1}}{b+1}\right)ds}=\frac{ct^{b}\exp\left(-\frac{ct^{b+1}}{b+1}\right)}{\exp\left(-\frac{ct^{b+1}}{b+1}\right)}=ct^{b},$$

which increases or decreases over time depending on the value of b. The corresponding mean function of the NHPP is given by

$$\Lambda \left(t\right)=\int_{0}^{t}\lambda \left(s|H\_s\right)ds=\int_{0}^{t}cs^{b}ds=c{\left(\frac{1}{b+1}\;s^{b+1}\right)}_{0}^{t}=\frac{ct^{b+1}}{b+1},$$

where c>0 and b>−1.

## Bivariate non-homogeneous Poisson process

Let {N(t),t≥0} be a Set of number of events that satisfy a NHPP whose occurrence times are distributed according to the Weibull distribution as described in subsection above with a conditional intensity function as given in Eq. (4),

$\lambda \left(t|H_{t}\right)=ct^{b},$Where c>0 and b>−1. The sequence of events generated by the process is classified into two types of events. Let $N_{1}\left(t\right)$ denote the number of type 1 events that occur in the observation interval time (0,t], and $N_{2}\left(t\right)$ denote the number of type 2 events that occur in the same period. If the probability that an event is type 1 within the interval (0,t] is p, then the following relationship holds.

Theorem 1 *Let* {N(t),t≥0} *be a NHPP with a conditional intensity function* $ct^{b}$*. Suppose that* $N_{1}\left(t\right)$ *denotes the number of type 1 events observed up to time* t*, where the probability of each event occurring at time* t *is* p*. Then the process* $\left\{N_{1}\left(t\right),\;t\geq 0\right\}$ *is also a NHPP with conditional intensity function given by* $pct^{b}$.

Proof:

Given that {N(t),t≥0} is a NHPP with a conditional intensity function $\lambda \left(t|H_{t}\right)=ct^{b}$, and suppose that within this process, certain events are classified as type 1 events.

We aim to show that the process $\left\{N_{1}\left(t\right),\;t\geq 0\right\}$ is also a NHPP with conditional intensity function $\lambda_{1}\left(t|H_{t}\right)=pct^{b}$, where p∈(0,1) is the probability that a given event is type 1.

Since $N_{1}\left(t\right)$ is a subset of an event from N(t), and because N(0)=0, then $N_{1}\left(0\right)=0$.

The increment of N(t) over disjoint time intervals is independent, so the process $N_{1}\left(t\right)$ inherits the independent increments.

Let $N_{1}\left(t,t+h\right)=N_{1}\left(t+h\right)-N_{1}\left(t\right)$ denote the number of type 1 events in the interval (t,t+h). Because $N_{1}\left(t\right)\leq N\left(t\right)$, we have

$$P\left(N_{1}\left(t,t+h\right)\geq 2\right)\leq P\left(N\left(t,t+h\right)\geq 2\right)=o\left(h\right),$$

so $P\left(N_{1}\left(t,t+h\right)\geq 2\right)=o\left(h\right)$. This confirms the Poisson property that at most one event occurs in a small interval.

Consider $P(N_{1}\left(t,t+h\right)=1)$. This probability can be decomposed

$$P\left(N_{1}\left(t,t+h\right)=1\right)=P\left(N_{1}\left(t,t+h\right)=1|N\left(t,t+h\right)=1\right)P\left(N\left(t,t+h\right)=1\right)$$

$$+P\left(N_{1}\left(t,t+h\right)=1|N\left(t,t+h\right)\geq 2\right)P\left(N\left(t,t+h\right)\geq 2\right)$$

$$=p\;P\left(N\left(t,t+h\right)=1\right)+\;P\left(N_{1}\left(t,t+h\right)=1|N\left(t,t+h\right)\geq 2\right)\;o\left(h\right)$$

$$=p\;ct^{b}\;h+\;o\left(h\right).$$

Hence the conditional intensity function for $N_{1}\left(t\right)$ is

$$\lambda_{1}\left(t|H_{t}\right)=\lim_{h\to 0}\frac{P\left(N_{1}\left(t,t+h\right)=1\right)}{h}=\lim_{h\to 0}\frac{p\;ct^{b}\;h+\;o\left(h\right)}{h}=p\;c\;t^{b}.$$

We conclude $N_{1}\left(t\right)$ is a NHPP with conditional intensity function $\lambda_{1}\left(t|H_{t}\right)=p\;ct^{b}\;h$.■

The NHPP with conditional intensity function $pct^{b}$ implies that the inter-event times of the type 1 event follow a Weibull distribution. Based on the derived intensity function, the probability density function of the inter-event time for type 1 events is given by

$$g\left(t\right)=pct^{b}\exp\left(-\frac{pct^{b}}{b+1}\right),$$

for t≥0. In a NHPP, it is possible to classify events into more than two distinct types. The process $\left\{N_{k}\left(t\right),\;t\geq 0\right\}$, representing the number of events of type k, k=1,2,⋯,m, is also a NHPP with conditional intensity function

$$\lambda_{k}\left(t|H_{t}\right)=p_{k}\;ct^{b}\;h,$$

where $p_{k}$ is the probability that an event of type k occurs within the interval (0,t] and $\sum_{k}p_{k}=1$.

Corollary 1 *Let* $\left\{N_{1}\left(t\right),\;t\geq 0\right\}$ *be a NHPP with intensity function* $\lambda_{1}\left(t\right)=pct^{b}$*. Then there exists a corresponding process* $\left\{N_{2}\left(t\right),\;t\geq 0\right\}$ *which is also a NHPP with intensity function* $\lambda_{2}\left(t\right)=\left(1-p\right)ct^{b}$.

Suppose that among a collection of event types (possibly more than two) only two specific types of events are of interest. If the occurrences of type 1 and type 2 events are independent of the entire process, then the joint probability distribution of $N_{1}\left(t\right)$ and $N_{2}\left(t\right)$ can be formulated as follows.

Corollary 2 *Let* $\left\{N_{1}\left(t\right),\;t\geq 0\right\}$ *and* $\left\{N_{2}\left(t\right),\;t\geq 0\right\}$ *be two independent NHPPs as mentioned* Corollary 1*. Then the joint process* $\left\{(N_{1}\left(t\right),N_{2}\left(t\right)),\;t\geq 0\right\}$ *forms a bivariate non-homogeneous Poisson distribution (BNHPP), with probability function given by*

$$P\left(n_{1},n_{2}\right)=\frac{1}{n_{1}!n_{2}!}\;{\left(\int_{0}^{t}pcs^{b}\right)}^{n_{1}}{\left(\int_{0}^{t}qcs^{b}\right)}^{n_{2}}\exp\left(-\int_{0}^{t}\left(p+q\right)cs^{b}ds\right),$$

where p and q are probabilities that a random event is classified as type 1 and type 2, respectively, and p+q≤1.

The BNHPP described in Corollary 2 is derived from two mutually independent point processes. Suppose that during the time period (0,T], the interval is partitioned into subintervals of the form $\left(t_{i-1},t_{i}\right]$, for i=1,2,⋯,n, each observed event is classified exclusively as a type 1 or type 2 event. The number of observed types 1 is $n_{1}$ and $n_{2}$ is the number of observed type 2 events.

**Fig. 1:**
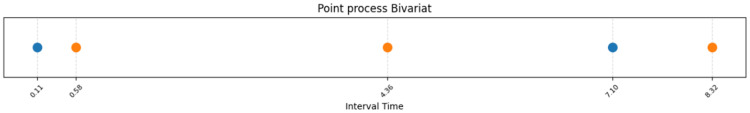
Bivariate Non-homogeneous Poisson process.

Fig. 1 illustrates a simulated realization of a BNHPP. Each point represents an event occurring within the time observation (0,10], with the two different types of events distinguished by blue and orange points. Fig. 1 displays the times of occurrence of events within the time observation. Total of five events are observed, resulting in six subintervals formed by partitioning the time axis at each event time.

## Bivariate point process model

In general, the probability model of a BNHPP, constructed from two independent NHPP that categorize events into two types, is given in Corollary 2 by the following joint probability function:

$$P\left(n_{1},n_{2}\right)=\frac{1}{n_{1}!n_{2}!}\;{\left(\int_{0}^{t}pcs^{b}\right)}^{n_{1}}{\left(\int_{0}^{t}qcs^{b}\right)}^{n_{2}}\exp\left(-\int_{0}^{t}\left(p+q\right)cs^{b}ds\right),$$

where $n_{1}$ denotes the number of type 1 events and $n_{2}$ denotes the number of type 2 events observed up to time t. At any given event time, the probability that the event belongs to type 1 is p, while the probability that it belongs to type 2 is q. The conditional intensity function governing the occurrence of events depends on two parameters, c which controls the baseline intensity, and b, which governs how the intensity changes over time.

Let the process $\left\{(N_{1}\left(t\right),N_{2}\left(t\right)),\;t\geq 0\right\}$ be observed over a fixed time interval (0,T]. During this observation period, suppose that a total of n random events occur at time points $t_{1},t_{2},\cdots ,\;t_{n}$ where each event corresponds to a type 1 or type 2 event and occurs at random. Among these n events, let $n_{1}$ denote the number of type 1 and $n_{2}$ denote the number of type 2 events, so that $n=n_{1}+n_{2}$.

Let the time observation (0,T] and suppose that the primary interest is in type 1 events. Consider a small subinterval of width h in $\left(t_{i-1},t_{i}\right]$, where h→0. The probability that exactly one event of type 1 occurs and that no event of type 2 occurs within this interval is denoted as $P\left(N_{1}\left(t_{i-1},t_{i}\right]=1,N_{2(t_{i-1},t_{i}]}=0\right)=P_{\left(t_{i-1},t_{i}\right]}\left(1,0\right)$,

$$P_{\left(t_{i-1},t_{i}\right]}\left(1,0\right)=\frac{1}{1!0!}\;\left(pc{\left(t_{i}\right)}^{b}h\right)\;{\left(qc{\left(t_{i}\right)}^{b}h\right)}^{0}\exp\left(-\int_{t_{i-1}}^{t_{i}}\left(p+q\right)ct^{b}dt\right)$$

$$=\frac{1}{1!0!}\;\left(pc{\left(t_{i}\right)}^{b}h\right)\;\exp\left(-\int_{t_{i-1}}^{t_{i}}\left(p+q\right)ct^{b}dt\right).$$

Similarly, the occurrence of exactly one type 2 event and no type 1 event, within a short time interval of length h in $\left(t_{i-1},t_{i}\right]$, can be expressed as $P\left(N_{1}\left(t_{i-1},t_{i}\right]=0,N_{2(t_{i-1},t_{i}]}=1\right)=P_{\left(t_{i-1},t_{i}\right]}\left(0,1\right),$

$$P_{\left(t_{i-1},t_{i}\right]}\left(0,1\right)=\frac{1}{0!1!}\;{\left(pc{\left(t_{i}\right)}^{b}h\right)}^{0}\;\left(qc{\left(t_{i}\right)}^{b}h\right)\exp\left(-\int_{t_{i-1}}^{t_{i}}\left(p+q\right)ct^{b}dt\right)$$

$$=\frac{1}{1!0!}\;\left(qc{\left(t_{i}\right)}^{b}h\right)\;\exp\left(-\int_{t_{i-1}}^{t_{i}}\left(p+q\right)ct^{b}dt\right).$$

For the observation time (0,T], each occurrence is monitored so that there are n event time points. This means that these time points divide the interval (0,T] into subintervals $\left(0,t_{1}\right],\;\left(t_{1},t_{2}\right],\;\cdots ,\left(t_{i-1},t_{i}\right],\cdots ,\left(t_{n-1},t_{n}\right]$, and $\left(t_{n},T\right]$. Fig. 1 provides an illustration of the observation period and the corresponding event time points. Each subinterval from $\left(0,t_{1}\right]$ to $\left(t_{n-1},t_{n}\right]$ contains exactly the event, no events occur in $\left(t_{n},\;T\right]$. Therefore, the number of type 1 events is $n_{1}$, and the number of type 2 events is $n_{2}$. Suppose $P_{\left(0,T\right]}\left(n_{1},n_{2}\right)=P\left(N_{1}\left(0,T\right]=n_{1},N_{2}\left(0,T\right]=n_{2}\right)$ denotes the probability of observing $n_{1}$ type 1 events and $n_{2}$ type events within (0,T], then

$$P_{\left(0,T\right]}\left(n_{1},n_{2}\right)=\prod_{i=1}^{n_{1}}P_{\left(t_{i-1},t_{i}\right]}\left(1,0\right)\;\prod_{i=1}^{n_{2}}P_{\left(t_{i-1},t_{i}\right]}\left(0,1\right)\;\times P_{\left(t_{n},T\right]}\left(0,0\right)$$

$$=\prod_{i=1}^{n_{1}}\left(pc{\left(t_{i}\right)}^{b}h\right)\;\exp\left(-\int_{t_{i-1}}^{t_{i}}\left(p+q\right)ct^{b}dt\right)$$

$$\times \prod_{i=1}^{n_{1}}\left(pc{\left(t_{i}\right)}^{b}h\right)\;\exp\left(-\int_{t_{i-1}}^{t_{i}}\left(p+q\right)ct^{b}dt\right)$$

$$\times \exp\left(-\int_{t_{n}}^{T}\left(p+q\right)ct^{b}dt\right)$$

$$={\left(pch\right)}^{n_{1}}{\left(qch\right)}^{n_{2}}{\left(\prod_{i=1}^{n_{1}+n_{2}}t_{i}\right)}^{b}\exp\left(-\int_{0}^{T}\left(p+q\right)ct^{b}dt\right).$$

The probability

(5) $$P_{\left(0,T\right]}\left(n_{1},n_{2}\right)={\left(pch\right)}^{n_{1}}{\left(qch\right)}^{n_{2}}{\left(\prod_{i=1}^{n_{1}+n_{2}}t_{i}\right)}^{b}\exp\left(-\int_{0}^{T}\left(p+q\right)ct^{b}dt\right),$$

describes the joint probability for two-point processes occurring within the time interval (0,T] and it is determined by the parameters p,q,c, and b. The form ${\left(\prod_{i=1}^{n_{1}+n_{2}}t_{i}\right)}^{b}$ reflects the strength of the influence of the event times modulated by the parameterb, where the parameter b serves as a time scaling factor in the model. Eq. (5) provides a model that captures the dynamics of events and the interaction between two concurrent point processes within a shared time interval.

## Likelihood function

Based on the probability of the BNHPP in Eq. (5), the likelihood function can be expressed as a function of the parameter vector θ=(p,q,c,b). The likelihood function requires all information obtained from the observation of BNHPP within the time interval (0,T] to estimate the parameter values the best fit the observed data.

$$L\left(\theta :n_{1},n_{2},\tau \right)=P_{\left(0,T\right]}\left(n_{1},n_{2}\right)={\left(pch\right)}^{n_{1}}{\left(qch\right)}^{n_{2}}{\left(\prod_{i=1}^{n}t_{i}\right)}^{b}\exp\left(-\frac{\left(p+q\right)cT^{b+1}}{b+1}\right),$$

where $\tau =(t_{1},t_{2},\cdots ,t_{n})$. The likelihood function is then divided by $h^{n_{1}+n_{2}}$,

$$L^{*}\left(\theta :n_{1},n_{2},\tau \right)={\left(pc\right)}^{n_{1}}{\left(qc\right)}^{n_{2}}{\left(\prod_{i=1}^{n}t_{i}\right)}^{b}\exp\left(-\frac{\left(p+q\right)cT^{b+1}}{b+1}\right).$$

For ease pf parameter estimation, the likelihood function is transformed into its log-likelihood form,

(6) $$l\left(\theta :n_{1},n_{2},\tau \right)=n_{1}\ln p+n_{2}\ln q+n\ln c+b\sum_{i=1}^{n}\ln t_{i}-\frac{\left(p+q\right)cT^{b+1}}{b+1}.$$

## Parameter estimation technique

Parameters estimation for the BNHPP model in Corollary 2 is carried out using the maximum likelihood method in Eq. (6). The sample data are assumed to come from observation recorded over a fixed observation period (0,T]. Consequently, the likelihood function is constructed on the basis of the observed event data.

The parameters of the BNHPP are estimated using the maximum likelihood estimation method. The optimal values of the parameters p,q,c, and b, which maximize Eq. (6), are obtained from $\frac{\partial l}{\partial p}=0$, $\frac{\partial l}{\partial q}=0$, $\frac{\partial l}{\partial c}=0$, and $\frac{\partial l}{\partial b}=0$. Then

$$\frac{\partial l}{\partial p}=\frac{n_{1}}{p}-\frac{cT^{b+1}}{b+1}=0;$$

$$\frac{\partial l}{\partial q}=\frac{n_{1}}{q}-\frac{cT^{b+1}}{b+1}=0;$$

$$\frac{\partial l}{\partial c}=\frac{n}{c}-\frac{\left(p+q\right)T^{b+1}}{b+1}=0;$$

$$\frac{\partial l}{\partial b}=\sum_{i=1}^{n}\ln t_{i}+\frac{cT^{b+1}}{{\left(b+1\right)}^{2}}-\frac{cT^{b+1}\ln T}{b+1}=0.$$

Thus, from the equations above, the estimators of the parameters are obtained as follows:

$$\hat{p}=\frac{n_{1}\left(b+1\right)}{cT^{b+1}};$$

$$\hat{q}=\frac{n_{2}\left(b+1\right)}{cT^{b+1}};$$

$$\hat{c}=\frac{n\left(b+1\right)}{\left(p+q\right)T^{b+1}}.$$

However, the estimator for the parameter b cannot be obtained in a closed from expression,

(7) $$\sum_{i=1}^{n}\ln t_{i}+\frac{cT^{b+1}}{{\left(b+1\right)}^{2}}-\frac{cT^{b+1}\ln T}{b+1}=0.$$

An explicit estimator for b can be obtained by substituting the estimator of c into Eq. (7).

$$\hat{b}=\frac{n}{n\ln T-\left(p+q\right)\sum_{i=1}^{n}\ln t_{i}}-1.$$

## Method validation

The proposed method is validated using two datasets. The first dataset generated through Python code, while the second consists of actual earthquake event data.

## Simulation of experiment two Poisson process

= To further the identifiability of parameter b, additional simulation experiments were conducted for b≠0. The simulation study is based on a parent NHPP with intensity function $\lambda \left(t\right)=ct^{b}$. The generated events are independently partitioned into two event types using probability p, resulting in two NHPPs.

**Fig. 2:**
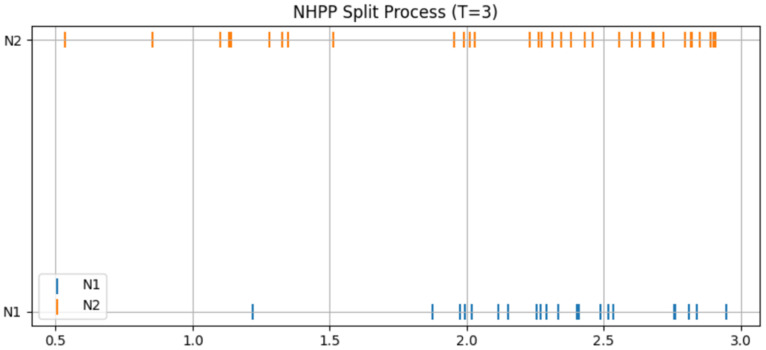
Simulation of a Bivariate Non-homogeneous Poisson Process.

Suppose that the observation time is given T=3, as shown in Fig. 2. The simulated data for p=0.4, c=3, and b=3 within the time interval (0,T]produced 56 events, with 21 events of type 1 and 35 of type 2.

Fig. 2 illustrates one realization of the simulation bivariate NHPP obtained by independently a parent NHPP with intensity function $\lambda \left(t\right)=ct^{b}$, where c=3, b=3, and p=0.4. The blue marks represent events assigned to process 1, whereas the red marks correspond to events assigned to process 2. Since the intensity function increases with time event occurrences become more frequent near the end of the observation interval time. Furthermore, the large number of events in Process 2 compared with Process 1 reflects the thinning probabilities 1−p=0.6 and p=0.4.

Based on the simulation results, the event times $t_{i}$ range from 0.53 to 2.95, showing that events are distributed over this time interval. Furthermore, the irregular pattern of the time interval between events reflects the characteristics of a NHPP. This variation illustrates the changing intensity of the event over time. Table 1 summarizes the quantities required for maximum likelihood estimation. A total of 56 events were generated from the parent NHPP with 21 and 35 events assigned to Process 1 and Process 2, respectively.

**Table 1:** Summary statistics obtained from the simulated bivariate NHPP.

Quantity	Value
Total number of events (N)	56
Number of events in Process 1 ($N_{1}$)	21
Number of events in Proces 2 ($N_{2}$)	35
ln⁡(T)	ln(3)≈1.099
$\sum_{i=1}^{N}\ln\left(t_{i}\right)$	41.999

If the data in Table 1 are used to estimate the parameters of a BNHPP with conditional intensity function $\lambda \left(t|H_{t}\right)=\left(p+q\right)ct^{b}$, the resulting estimators are as follows:

$$\hat{p}=\frac{n_{1}}{n_{1}+n_{2}}=\frac{21}{56}=0.375;$$

$$\hat{q}=\frac{n_{2}}{n_{1}+n_{2}}=\frac{35}{56}=0.625;$$

$$\hat{b}=\frac{n_{1}+n_{2}}{\left(n_{1}+n_{2}\right)\ln T-\sum_{i=1}^{n_{1}+n_{2}}\ln t_{i}}-1=\frac{56}{56\ln3-41.999}-1=1.868;$$

$$\hat{c}=\frac{\left(n_{1}+n_{2}\right)\left(\hat{b}+1\right)}{T^{\hat{b}+1}}=\frac{56\left(1.868+1\right)}{3^{\wedge }\left(1.868+1\right)}=6.875.$$

Thus, the estimated conditional intensity function is given by

$$\hat{\lambda }\left(t|H_{t}\right)=\hat{c}\;t^{\hat{b}}=6.875\;t^{1.868}.$$

Simulation were performed underdifferent observation times T=3,T=5,T=10,T=15, and T=20, to evaluate the performance of the proposed estimation method. The resulting summary statistics are presented in Table 2, whereas Table 3 reports the corresponding maximum likelihood estimates of the model parameters.

**Table 2:** Summary statistics from generated data.

T	$n_{1}$	$n_{2}$	n	ln⁡(T)	$\sum_{i=1}^{N}\ln\left(t_{i}\right)$
3	21	35	56	1.098612	41.9988
5	176	250	426	1.609438	573.8176
10	2993	4535	7528	2.302585	15,465.28
15	15,442	22,778	38,220	2.70805	93,984.45
20	47,919	71,385	119,304	2.995732	327,614.3

**Table 3:** Parameter estimation of the intensity function for different observation T.

T	$\hat{p}$	$\hat{q}$	$\hat{b}$	$\hat{c}$	$\lambda \left(t|H_{t}\right)=\hat{c}t^{\hat{b}}$
3	0.375	0.625	1.86834	6.874997	$6.874997\;t^{1.86834}$
5	0.413146	0.586854	2.810274	3.524509	$3.524509\;t^{2.810274}$
10	0.397582	0.602418	3.028733	2.838671	$2.838671\;t^{3.028733}$
15	0.404029	0.595971	3.015874	2.90427	$2.90427\;t^{3.015874}$
20	0.401655	0.598345	3.005025	2.941728	$2.941728\;t^{3.005025}$
**True**	**0.4**	**0.6**	**3**	**3**	$\lambda \left(t|H_{t}\right)=3t^{3}$

**Fig. 3:**
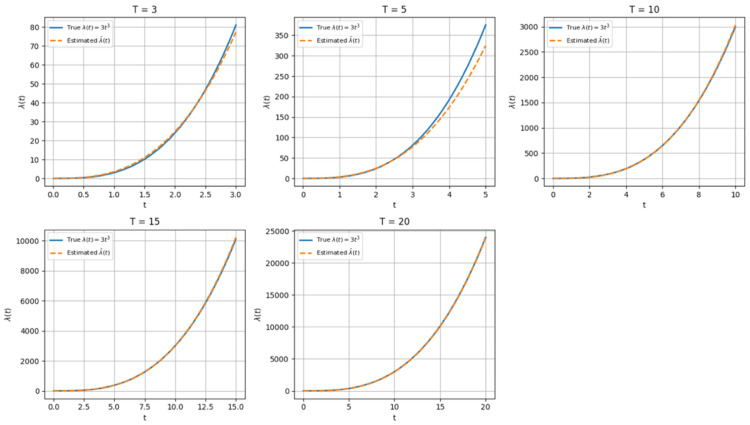
Intensity function plots under varying observation time T.

The comparison of conditional intensity function graphs for different observation times can be seen in Fig. 3.

The graph in Fig. 3 compares the true intensity function $\lambda \left(t\right)=ct^{b}=3t^{3}$ with the estimated intensity function corresponding to different observation time. As the observation period becomes longer, the estimated curves gradually approach the true intensity function. This improvement reflects the increasing amount of information available from the observed events, allowing the proposed maximum likelihood estimation to recover the underlying intensity structure more accurately.

## Goodness of fit assesment using the time rescaling theorem

To evaluate the adequacy of the proposed Bivariate NHPP model, a godness of fit assesment based on time rescaling theorem was performed. If the intensity function is correctly specified, the transformed inter event times should form an independent and identically distributed sample from an exponential distribution with rate one denoted by Exp(λ=1). For the intensity function

$$\lambda \left(t\right)=ct^{b},$$

the transformed inter-event times are computed as

$$\tau_{i}=\int_{t_{i-1}}^{t_{i}}\lambda \left(s\right)ds=\int_{t_{i-1}}^{t_{i}}cs^{b}ds=\frac{c}{b+1}\left(t_{i}^{b+1}-t_{i-1}^{b+1}\right),$$

with $t_{0}=0$.

Under a correctly specified model, the transformed values $\tau_{i}$ should follow Exp(λ=1). Therefore, the goodness of fit of the proposed model was evaluated by QQ-plot and a Kolmogorov-Smirnov test.

**Fig. 4:**
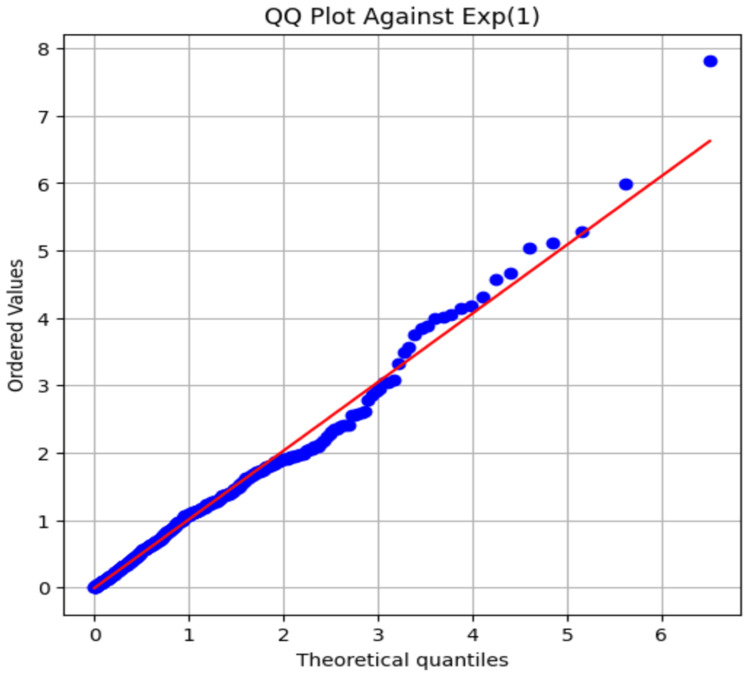
QQ-Plot of the rescaling time from the simulated BNHPP Data with *T *= 5.

Fig. 4 shows that the transformed inter-event times closely follow the theoretical quantiles of the exponential distribution with mean 1. This visual agreement is further supported by the Kolmogorov-Smirnov test. The Kolmogorov-Smirnov test produced a statistic of D=0.0119 with a p-value of 0.2540. Therefore, the null hypothesis that the transformed inter-event times follow an Exp(1) distribution cannot be rejected at the 5% significance level.

## Case study

The BNHPP model will be applied to explore the point process structure of earthquake events with magnitude of 5.0 or greater that may occur on Sulawesi island. Due to the limited number of such high-magnitude earthquakes -averaging only 3.5 events per month conducting a NHPP analysis solely on these events is not adequate. Therefore, the entire set of earthquake events will be used under the assumption that the data can be partitioned into two types of events: earthquake with magnitude below 5.0 and those with magnitude of 5.0 or above. The data set covers the period from January 2018 to December 2020 and is sourced from the Meteorology, Climatology, and Geophysics Agency (BMKG), region IV Makassar.

Table 4 presents the monthly counts of earthquake events in the two classification during the years 2018 to 2020, which further divide into three observation periods; 2018, 2019, and 2020. According to Table 3, the highest number of earthquakes occurred in November 2018, with a total 1401 events. In contrast, August 2019 recorded the lowest number of events, with only 73 events. During the entire observation period from 2018 to 2020, there were 8 months in which no earthquakes of magnitude 5.0 or greater were recorded.

**Table 4:** Data on the number of earthquakes that occurred on Sulawesi Island from January 2018 to December 2020.

Month	Mag 1–5	Mag 5- up	Month	Mag 1–5	Mag 5- up	Month	Mag 1–5	Mag 5- up
Jan-18	193	3	Jan-19	195	0	Jan-20	136	3
Feb-18	161	3	Feb-19	146	4	Feb-20	159	4
Mar-18	261	8	Mar-19	132	1	Mar-20	266	2
Apr-18	168	1	Apr-19	72	2	Apr-20	247	1
May-18	138	2	May-19	94	4	May-20	226	0
Jun-18	148	0	Jun-19	160	2	Jun-20	239	7
Jul-18	183	2	Jul-19	108	0	Jul-20	204	0
Aug-18	163	0	Aug-19	72	1	Aug-20	191	6
Sep-18	518	32	Sep-19	99	0	Sep-20	194	1
Oct-18	940	8	Oct-19	74	2	Oct-20	318	7
Nov-18	1393	8	Nov-19	109	2	Nov-20	253	1
Dec-18	431	3	Dec-19	128	0	Dec-20	214	6

Each earthquakes event in this data set was recorded by BMKG on the basis of its exact time occurrence, incluiding the date and timestamp. Therefore the data are naturally represented as temporal point process, where each recorded time corresponds to a single earthquake event. Although monthly aggregated counts are represented in Table 4. To provide a concise summary of dataset, the maximum likelihood estimationwas performed using the original event times. Tereby preserving the full temporal resolution of the data.

On the basis of its exact time occurence, incuiding the date and time, making it a point process where each time point represents a singleseismic event. Let $N_{1}$is the number of earthquakes with a magnitude of 5.0 or greater (type 1) and $N_{2}$ is the number of other event (type 2). Then the form of the probability function of the bivariate point process over each observation period (0,T] is the following

$$f\left(n_{1},n_{2};p,c,b\right)=p^{n_{1}}{\left(1-p\right)}^{n_{2}}{\left(ch\right)}^{n_{1}+n_{2}}{\left(t_{1}t_{2}\cdots t_{n_{1}+n_{2}}\right)}^{b}\exp\left(-\frac{c\;T^{b+1}}{b+1}\right),$$

where $t_{1},t_{2},\cdots ,\;t_{n_{1}+n_{2}}$ are the occurrence times of type 1 and type 2, h→0 represents the infinitesimal time interval around the occurrence time $t_{i}$. The form of the log-likelihood function, based on Eq. (6) is

$$l\left(p,c,b\right)=n_{1}\ln p+n_{2}\ln\left(1-p\right)+\left(n_{1}+n_{2}\right)\ln c+b\sum_{i}^{n_{1}+n_{2}}\ln t_{i}-\frac{c\;T^{b+1}}{b+1}.$$

Parameter estimates obtained using the maximum likelihood method are as follows

$$\frac{\partial l}{\partial p}=\frac{n_{1}}{p}-\frac{n_{2}}{1-p}=0\Rightarrow \hat{p}=\frac{n_{1}}{n_{1}+n_{2}}$$

$$\frac{\partial l}{\partial c}=\frac{n_{1}+n_{2}}{c}-\frac{T^{b+1}}{b+1}=0\Rightarrow \hat{c}=\frac{\left(n_{1}+n_{2}\right)\;\left(b+1\right)}{T^{b+1}}$$

$$\frac{\partial l}{\partial b}=\sum_{i}^{n_{1}+n_{2}}\ln t_{i}+\frac{cT^{b+1}}{{\left(b+1\right)}^{2}}-\frac{c\;T^{b+1}\ln T}{b+1}\;=0\;\Rightarrow \hat{b}=\frac{\left(n_{1}+n_{2}\right)+\sum_{i=1}^{n_{1}+n_{2}}\ln t_{i}-\left(n_{1}+n_{2}\right)\ln T}{\left(n_{1}+n_{2}\right)\ln T-\sum_{i=1}^{n_{1}+n_{2}}\ln t_{i}\;}.$$

Suppose that the conditional intensity function is to be examined for years 2018, 2019, and 2020 based on the daily occurrence times of earthquakes. With the observation time T set to one year, there are three observation periods: $T_{2018}=T_{2019}=364$ days and $T_{2020}=365$ days. Table 4 presents the maximum likelihood estimates for the three time periods (Table 5).

**Table 5:** Yearly estimates of earthquake intensity function parameters based on daily data.

Year	Days	$\hat{c}$	$\hat{b}$	$\hat{p}$	$c^{*}=\hat{p}\;\hat{c}$
2018	364	0.3366	0.7123	0.0147	0.0049
2019	364	9.9814	−0.1984	0.0128	0.1277
2020	365	3.3218	0.1609	0.0144	0.0480

The daily earthquake occurrence data for 2018, 2019, and 2020 were analyzed using an conditional intensity function $\lambda \left(t|H_{t}\right)=c\;t^{b}$, where t represents the time in days, and the parameters c and b characterize the temporal behavior of the seismic activity. To specifically model the occurrence of earthquake type 1, the function was extended to $\lambda_{1}\left(t|H_{t}\right)=pc\;t^{\wedge }b$, where p denotes the probability that a given earthquake of magnitude 5 or greater is likely to occur.

In 2018, the estimated value of b=0.7123 indicates a relatively strong increase trend in eartquake frequency over time. This suggest that the intensity of seismic activity, including earthquake type 1, was more concentrated toward the end of the years, indicating a relatively elevated risk of significant seismic events in the surrounding Sulawesi island.

In contrast, in 2019, the negative value b=−0.1984 implies a downward trend in earthquake activity throughout the year 2019. The large estimated value of c=9.9814 reflects the high initial seismic intensity, which then diminished over time. The probability of earthquake type 1 was the lowest at p=0.0128, suggesting a lower overal risk.

For 2020, the parameter b=0.1609 indicates a modest upward trend in seismic intensity over time. The value of c=3.3218 suggests a moderate initial activity that gradually increased. The probability of earthquake type 1, p=0.0144

Was close to that of 2018, implying a relatively consistent level of risk of significant events throughout the year 2020.

The proposed model assumes independent event occurrences conditional on the intensity function. However, earthquake occurrences may exhibit aftershock sequences and self-exciting behavior that are not accomodated by the present formulation. Extensions incorporating event interaction structures may therefore provide improved goodness of fit.

## Limitations

The proposed bivariate model is obtained through independent thinning of a parent NHPP. Consequently, the occurrence of one event type does not directly affect the conditional intensity of other type. While this assumption simplifies inference and facilitates closed-form likelihood construction, it may not fully capture triggering mechanism observed in some applications, such as earthquake sequences. Extensions incorporating cross-excitation effect remain a topic for future investigation.

## Ethics statements

None are applicable to this work.

## Acknowledgments

The authors acknowledge the funding support for this research provided by the internal grant of the Faculty of Mathematics and Natural Sciences. The authors sincerely thank the experts for their valuable and constructive suggestions to improve this paper.

## Supplementary material *and/or* additional information [OPTIONAL]

Data will be made available on request.

## CRediT authorship contribution statement

**Andi Kresna Jaya:** Conceptualization, Methodology, Writing – original draft, Data curation. **Nurtiti Sunusi:** Supervision, Writing – review & editing. **Erna Tri Herdiani:** Supervision, Writing – review & editing.

## Declaration of interests

The authors declare that they have no known competing financial interests or personal relationships that could have appeared to influence the work reported in this paper.

## Data Availability

Data will be made available on request.
